# Evidence hierarchies relating to hand surgery: current status and improvement. A bibliometric analysis study

**DOI:** 10.1590/1516-3180.2017.0146260617

**Published:** 2017-11-17

**Authors:** Thaís Silva Barroso, Marcelo Cortês Cavalcante, João Baptista Gomes dos Santos, João Carlos Belloti, Flávio Faloppa, Vinícius Ynoe de Moraes

**Affiliations:** I MD. Hand Surgery Resident, Department of Orthopedics and Traumatology, Escola Paulista de Medicina - Universidade Federal de São Paulo (EPM-UNIFESP), São Paulo (SP), Brazil.; II MD. Resident in Orthopedic Surgery, Department of Orthopedics and Traumatology, Escola Paulista de Medicina - Universidade Federal de São Paulo (EPM-UNIFESP), São Paulo (SP), Brazil.; III MD, PhD. Adjunct Professor, Department of Orthopedics and Traumatology, Escola Paulista de Medicina - Universidade Federal de São Paulo (EPM-UNIFESP), São Paulo (SP), Brazil.; IV MD, PhD. Full Professor, Department of Orthopedics and Traumatology, Escola Paulista de Medicina - Universidade Federal de São Paulo (EPM-UNIFESP), São Paulo (SP), Brazil.; V MD, PhD. Orthopedic Surgeon, Department of Orthopedics and Traumatology, Escola Paulista de Medicina - Universidade Federal de São Paulo (EPM-UNIFESP), São Paulo (SP), Brazil.

**Keywords:** Hand, Orthopedics, Evidence-based medicine

## Abstract

**BACKGROUND:**

Hierarchy of evidence is an important measurement for assessing quality of literature. Information regarding quality of evidence within the Brazilian hand surgery setting is sparse, especially regarding whether research has improved in either quality or quantity. This study aimed to identify and classify hand surgery studies published in the two most important Brazilian orthopedics journals based on hierarchy of evidence, with comparisons with previously published data.

**DESIGN AND SETTING:**

Bibliometric analysis study performed in a federal university.

**METHODS:**

Two independent researchers conducted an electronic database search for hand surgery studies published between 2010 and 2016 in Acta Ortopédica Brasileira and Revista Brasileira de Ortopedia. Eligible studies were subsequently classified according to methodological design, based on the Haynes pyramid model (HP) and the JBJS/AAOS levels of evidence and grades of recommendations (LOR). Qualitative and quantitative data were gathered regarding all studies. Previous data were considered to assess whether the proportion of high-quality studies had improved over time (2000-2009 versus 2010-2016).

**RESULTS:**

The final analysis included 123 studies, mostly originating from the southeastern region (78.8%) and private institutions (65%), with self-funding (91.8%). Methodological assessment showed that 15.4% were classified as level I/II using HP and 16.4% using LOR. No significant difference in proportions of high-quality studies was found between the two periods of time assessed (5% versus 12%; P = 0.13).

**CONCLUSION:**

Approximately 15% of hand surgery studies published in two major Brazilian journals were likely to be classified as high-quality through two different systems. Moreover, no trend towards quality-of-evidence improvement was found over the last 15 years.

## INTRODUCTION

The systematic approach of evidence-based medicine involves critical appraisal and stratification into levels of evidence^1^^,^^2^^,^^3^ as a first step. Classification of research considering its internal validity is important in translating research results into clinical practice.^1^^,^^2^

In this regard, stratification of evidence is the key to distinguishing robust high-quality research from biased or low-quality research. Stratification is demanded, given that the number of published studies in the literature is increasing year by year.^4^ Poolman indicated that higher quality research is linked to better reporting, which relates to trustworthiness and applicability.^5^

As a basic principle, researchers and practitioners should consider the best evidence available, in making health-related decisions. However, it is often not easy to distinguish good from poorly performed research. Thus, systematic reviews (SRs) are an important tool for combining and summarizing relevant previously published studies.^2^^,^^4^ Most SRs only consider level I and sometimes level II studies as eligible for data synthesis. Therefore, only highly unbiased studies are eligible for inclusion and final analysis.

In the setting of hand surgery, although there has been an absolute increase in research production, little is known about the quality of the evidence generated. A previous study suggested that higher levels of evidence are related to higher applicability within clinical, academic and educational scenarios.^6^

One Brazilian study from the early 2000s assessed hand surgery studies and demonstrated that only a low proportion provided level I and II evidence, accounting for less than 10% of all the studies analyzed.^7^ These data^7^ are in accordance with other findings in other settings.^8^ Bibliometric analyses, as performed in these two studies,^7^^,^^8^ are important because they can potentially have an impact on research policies and academic actions and can pinpoint unnecessary or unethical studies.^7^^,^^9^

### Hypothesis

The hypothesis for the present investigation was that recent studies have improved in terms of scientific methodology, thus moving towards a proportional increase in the numbers of level I and II studies produced.

## OBJECTIVES

This study aimed to:

1. Identify hand surgery studies published over the last five years (2010-2016) in the two main Brazilian orthopedics journals: Acta Ortopédica Brasileira (AOB) and Revista Brasileira de Ortopedia (RBO).
2. Classify the types of study and levels of evidence according to evidence-based medicine hierarchies.
3. Compare findings from two different periods (2000-2009 versus 2010-2016) within the same journal using the same methodology.

## METHODS

This study was approved by the local ethics committee of our institution (Universidade Federal de São Paulo, UNIFESP) under the number CAAE 60911016.8.0000.5505. The methodology used for this study was similar to that used in the senior author’s previous publication.^7^

### Search strategy

Using the specific web databases of the two journals (AOB and RBO), two researchers (M.C. and T.B.) independently evaluated all studies published between January 1, 2010, and December 31, 2016. These two prominent journals were chosen since they are national-level journals in Brazil that have an orthopedics scope and are indexed in international research databases (SciELO and MEDLINE).

Studies were initially screened based on their titles and were classified as eligible, potentially eligible or not eligible. The initial inclusion criteria included the presence of the following themes in the titles/abstracts: hand and wrist fractures, peripheral nerve lesions and vascular lesions in the upper limbs, nail bed lesions, brachial plexus lesions, muscle tendon lesions, upper-limb skin coverage, microsurgery, upper-limb pain syndromes, upper-limb congenital malformations, and anatomical and experimental studies. From the methodological perspective, narrative reviews, economic appraisal studies and experimental studies *in vitro* or on animals were excluded.

After this initial screening, eligible and potentially eligible studies were assessed: first using the abstracts and then the full-text articles. These studies were evaluated by the two examiners, who subsequently categorized them according to study type and level^10^ of evidence, using two different approaches: the Haynes pyramid of evidence (HP) and the JBJS/AAOS Evidence-Based Practice Committee guideline - levels of evidence and grades of recommendations (LOR).^11^ Stratification was conducted after reading the full text of all eligible studies. Any disagreements were resolved by a third evaluator (V.Y.M.).

### Haynes pyramid of evidence

We considered that systematic reviews of randomized clinical trials provided evidence at level I; randomized clinical trials, level II; cohort and case-control studies, level III; case series, level IV; and case reports, level V.

### JBJS/AAOS Evidence-Based Practice Committee guideline

This guideline, produced jointly by the Journal of Bone and Joint Surgery (JBJS) and the American Academy of Orthopaedic Surgeons (AAOS), is an improved, robust and detailed version of the previous HP stratification. Its levels of evidence are classified as follows:

#### Level I

Randomized controlled trial (RCT): a study in which patients are randomly assigned to the treatment or control group and are followed prospectively; or a meta-analysis on randomized trials with homogeneous results.

#### Level II

Poorly designed RCT: follow up data on less than 80% of patients.

Prospective cohort study (therapeutic): a study in which patient groups are separated non-randomly according to exposure or treatment, with exposure occurring after the study started.

Meta-analysis on Level II studies.

#### Level III

Retrospective cohort study: a study in which patient groups are separated non-randomly according to exposure or treatment, with exposure occurring before the study started.

Case-control study: a study in which patient groups are separated according to the current presence or absence of disease and examined for the prior exposure of interest.

Meta-analysis on Level III studies.

#### Level IV

Case series: a report on multiple patients with the same treatment, but no control group or comparison group.

#### Level V

Case report (a report on a single case), expert opinion or personal observation.

For all the studies ultimately included, we obtained information regarding the journal (AOB or RBO); geographic location of the study (south, southeast or north plus northeast plus center-west of Brazil); number of authors; and funding. Case reports were excluded from the analysis.

### Statistical analysis

Descriptive statistics consisting of the mean (following by standard deviation) and proportions were produced. Fisher’s F test was used to evaluate the proportions between the two periods of assessment. We considered P-values < 0.05 to be statistically significant.

## RESULTS

### Study characteristics

A total of 1200 papers in the journals’ databases were screened. From these, 123 (10.2%) were eligible for the current study. Sixty-three were retrieved from Acta Ortopédica Brasileira (51.2%) and 60 (48.8%) from Revista Brasileira de Ortopedia. The agreement between the observers for inclusion of the studies was 98.8%. Table 1 depicts the results from the data retrieved covering the period 2010-2016 and historical data from the previous study (2000-2009) on the same subject and journals.^7^ The data distribution in the two periods did not show any differences in the assessed outcomes between these periods (2000-2009 versus 2010-2016), since the confidence intervals overlapped for all relevant data.

**Table 1.**
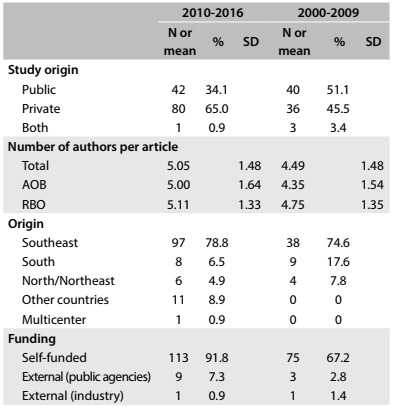
Study characteristics - qualitative and quantitative data

SD = standard deviation; AOB = Acta Ortopédica Brasileira; RBO = Revista Brasileira de Ortopedia.

Most studies were from private institutions (65%), were self-funded (91.8%) and were conducted in Brazil’s southeastern region (78.8%). The distribution of the studies conducted in other countries (12 studies) was: Turkey (4 studies), Portugal (3 studies) and others (5 studies; one each from China, Colombia, Uruguay, Italy and a multicenter study).

### Evidence hierarchy assessment

#### Haynes pyramid of evidence

**Graph 1.**
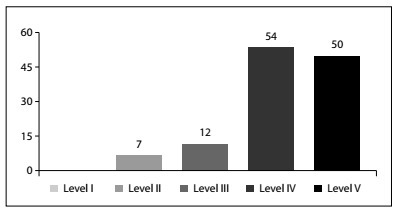
Distribution of studies as proposed using the Haynes model.

Considering the standard classification as published by Haynes, most of the studies were considered to present evidence at level IV/V. No systematic reviews of randomized trials (RCTs) on hand surgery were recognized. However, we found 7 RCTs and 12 case-control/cohort studies, which encompassed 15.4% of the total number of studies considered, as shown in Graph 1.

#### JBJS/AAOS Evidence-Based Practice Committee Guideline

**Graph 2.**
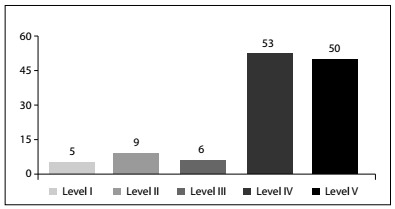
Distribution of studies according to the JBJS/AAOS Evidence-Based Practice Committee Guideline.

The more comprehensive criteria proposed by the Journal of Bone and Joint Surgery showed a similar trend. Level I, II and III studies encompassed 16.4% of the total number of studies assessed. As occurred with the HP assessment, the majority of the studies were level IV and V. Graph 2 shows the distribution of the studies according to this classification.

#### Comparison with historical data from previous study:2000-2009 versus 2010-2016

In our previous report (2000-2009), we recognized 83 studies and only four were considered as presenting level I or II according to HP. There were no statistical differences (Fisher’s F test, P = 0.13) in the proportion of published studies with level I or II evidence between 2000-2009 (4/83) and 2010-2016 (14/123).

## DISCUSSION

Our study characterized the current panorama of hand surgery research published in Brazilian journals. Two different criteria were used to classify these studies. We first used the extended pyramid model proposed by Haynes in 2006. Each of these levels should build systematically from lower levels and provide substantially more useful information for guiding clinical decision-making.^10^ Secondly, the JBJS/AAOS Evidence-Based Practice Committee Guideline.^11^ This was created by a task force of representatives from the AAOS Evidence-Based Practice Committee and the Journal of Bone and Joint Surgery, with the aim of providing the best answers to questions about interventions, in a timely manner. As far as we know, this was the first study to include both evidence hierarchy criteria in the same investigation.

We demonstrated that approximately 15% of the available research may be considered to present high quality-evidence (level I or II). In comparison with our previous analysis (2000 to 2009), a trend towards improvement of evidence was identified, although this was not statistically significant. Our findings reflect the challenge of conducting high-quality studies relating to hand surgery, such as blinded RCTs.

Classifying studies within the hierarchy of evidence is important as a first step. However, some published data have proven that RCTs may be prone to a great variety of systematic errors, which means that analysis on the internal validity of each study is an essential measurement for assessing its quality.^12^ Bias assessment is another means of rating research and may be standardized using specific tools. However, to our knowledge, there is no consensus in the literature regarding the application of such assessments.^13^

Recent research conducted on papers published in other journals, such as Plastic and Reconstruction Surgery, Journal of Plastic, Reconstructive and Aesthetic Surgery, Journal of Hand Surgery - European Volume, Journal of Hand Surgery - American Volume, Journal of Bone & Joint Surgery and Bone & Joint Journal, has demonstrated similar low rates of high-quality studies (11.2%). This shows that the data regarding hand surgery are in line with data from other specialties.^14^

Another study reviewed all online articles published in 2010 in The Spine Journal (TSJ), Spine, European Spine Journal (ESJ), Journal of Neurosurgery: Spine (JNS) and Journal of Spinal Disorders and Techniques (JSDT). It found that 27.9% of the articles were of high quality and that spinal surgery journals with higher impact factors contained higher proportions of studies of better quality.^15^

Research on the neurosurgical literature from 2009 to 2010 demonstrated that only 10.3% of the studies were of high quality. Only 1 in 10 of the studies was classified as presenting a high level of evidence.^16^

Research in the palliative medicine literature has shown that there was an increase in the proportion of studies presenting a high level of evidence among all published articles, from 0.08% in 1970 to 0.38% in 2005. However, it does not show the quality of the studies, only the quantity.^17^

Finally, our findings may not reflect the current status of Brazilian hand surgery research. We believe that the quantity of RCTs may have been underestimated, given that relevant high-quality research tends to be published in high-impact journals, with greater visibility and academic impact. Broader analysis on this subject might explore these phenomena in the future.

## CONCLUSIONS

Approximately 15% of hand surgery studies published in two major Brazilian journals are likely to be classified as high quality through two different classification systems. In addition, no trend towards improvement of the quality of evidence over the last 15 years was found.
